# Ain’t I Supposed to Be Doing Something?: Identifying and Discussing Cognitive Issues in Lupus

**DOI:** 10.1093/geroni/igaa057.1462

**Published:** 2020-12-16

**Authors:** Ann Vandenberg, Cristina Drenkard, Felicia Goldstein, Charmayne Dunlop-Thomas, S Sam Lim, C Barrett Bowling, Laura Plantinga

**Affiliations:** 1 Emory University School of Medicine, Atlanta, Georgia, United States; 2 Duke University Medical Center, Durham, North Carolina, United States; 3 Emory University, Atlanta, Georgia, United States

## Abstract

Lupus is a complex chronic disease associated with a high prevalence geriatric syndromes and reduced cognitive functioning, consistent with accelerated aging. Although patients with lupus commonly report cognitive symptoms or “lupus fog,” cognitive assessment is not routine and little is known about day-to-day cognitive problems and their effect on chronic disease management. As part of a pilot exploring the use of a cognitive functioning report prototype for shared decision making in clinical encounters (Approaches to Positive Patient-Centered Experiences of Aging in Lupus study), we conducted four focus groups, two with lupus patients (n=18) and two with lupus providers (physicians and nurses; n=9) addressing cognition. We compared how the NIH Toolbox fluid cognition domains (episodic (retrospective) memory, working memory, processing speed, attention and inhibitory control, cognitive flexibility, and overall fluid cognition) matched with patient- and provider-identified cognitive problems and needs. In open questioning using a standard interview guide, patients identified all domains with rich experiential examples; providers identified fewer domains with less detail. An unanticipated additional domain was prospective memory, problems in remembering future actions, whether in the near future (e.g., forgetting what they needed to do on entering a room) or in the not-so-near future (e.g., forgetting about a medical appointment). Technological aids (e.g., smartphone alerts) were mentioned by some patients, but not providers, and represent a potential opportunity for medical care. Providers envisioned using cognitive assessment to distinguish cognitive issues (e.g., forgetting to take medication) from other problems (e.g., medication non-adherence) in order to tailor chronic disease management messaging.

